# Atypical Ductal Hyperplasia Presenting as Vulvar Mass

**DOI:** 10.7759/cureus.96592

**Published:** 2025-11-11

**Authors:** Emmanuel Garrido-Cortes, Yolcar Chamorro, Niloofar Nasseri-Nik, Ana C Sandoval Leon

**Affiliations:** 1 Research, Alabama College of Osteopathic Medicine, Dothan, USA; 2 Oncology, Miami Cancer Institute, Miami, USA; 3 Pathology, Baptist Health South Florida, Miami, USA

**Keywords:** atypical ductal hyperplasia, breast carcinoma, cancer screening, ectopic breast tissue, rare breast pathology, vulva

## Abstract

Ectopic breast tissue (EBT) is a rare condition most frequently located in the axilla and only rarely in the vulva. It can undergo similar benign or malignant changes as normal breast tissue, including rare instances of atypical ductal hyperplasia (ADH). We report a 63-year-old woman with ectopic ADH in the right labia majora, initially misdiagnosed as a benign cyst. Histopathology confirmed EBT with ADH, and subsequent re-excision showed no residual disease. Immunohistochemistry supported the mammary origin. This case highlights the importance of histological examination for vulvar lesions suspected of EBT and underscores the need for guidelines on managing these rare occurrences.

## Introduction

Ectopic breast tissue (EBT) is a rare finding that occurs because of residual tissue along the embryonic milk lines, which extend from the axilla to the perineum [1-3]. The most frequent location for ectopic mammary tissue is the axilla and, more rarely, the vulva [4,5]. The predominant pathology found in EBT is benign tumors, primarily fibroadenomas. Breast carcinoma arising within EBT is uncommon, with an incidence of approximately 0.3%-0.6% among women [1]. The first case report of EBT of the vulva was reported by Hartung in 1872. In the report, a 30-year-old woman was described to have developed a fully formed mammary gland of the left labia majora [6]. Several case reports thereafter have discussed the extremely rare occurrence of both benign and carcinogenic ectopic breast tissue; however, documentation of ectopic atypical ductal hyperplasia (ADH) in the vulva appears to be lacking in the English medical literature. ADH bears similarities to low-grade ductal carcinoma in situ (DCIS), characterized by cytonuclear and architectural abnormalities; however, it differs by either involving only parts of the ducts or being smaller in size compared to what is typically diagnosed as DCIS [7]. While ADH is considered benign, it is categorized as a high-risk precursor condition given its potential to develop into DCIS, which can further progress to invasive carcinoma [8]. In this report, we present a patient who developed ectopic ADH in the right labia majora that was initially believed to be a benign cyst but was later diagnosed as EBT with foci of ADH on histopathology.

This case report was previously presented as a poster at the 2024 San Antonio Breast Cancer Symposium on December 13, 2024.

## Case presentation

**Figure 1:**
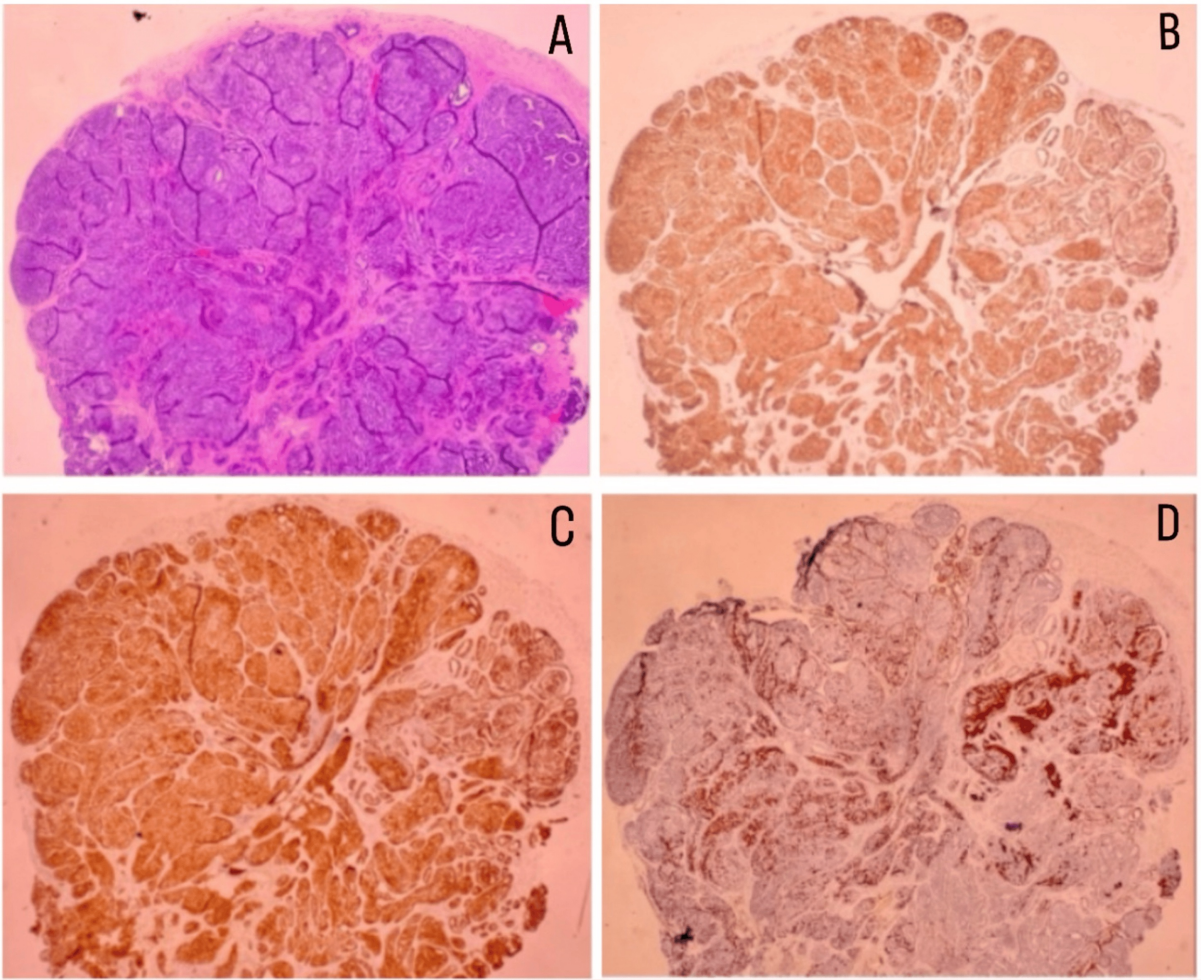
Histological and immunohistochemical features of the right labial mass (A) Low-power view of the right labial mass, demonstrating the overall architecture. (B) GATA-3 stain, supporting mammary differentiation. (C) ER stain showing diffuse strong positivity (3+/3). (D) CK5 stain, partially negative with diffuse and strong staining for ER, supporting the diagnosis of ADH. GATA3: GATA-binding protein 3, ER: estrogen receptor, CK5: cytokeratin 5, ADH: atypical ductal hyperplasia

A 63-year-old woman had a lump in her labia for several years. She had a past medical history of endometrial polyps and a family history of breast cancer in her mother and her sister. On a physical examination, she had a 5 mm cystic lesion in the right labia. She was evaluated by her gynecologist for endometrial polyps, and she asked to remove the vulvar cystic lesion during the procedure. Pathology of the cystic vulvar lesion revealed EBT with foci of ADH (Figure 1A). The mass was immunoreactive for GATA-binding protein 3 (GATA3) (Figure 1B), estrogen receptors (Figure 1C), and progesterone receptors. Myoepithelial cells were identified via p63/SMM staining. The diagnosis of ADH was supported by the pattern of staining for cytokeratin 5 (CK5) (Figure 1D) and estrogen receptors. A stain for paired-box 8 (PAX8) and erythroblast transformation-specific regulated gene 1 (ERG) was negative. The mass was incompletely excised, and additional surgery was recommended. Upon re-excision, no residual ADH or EBT were seen.

Due to these findings, she was referred to the Breast Cancer Prevention Clinic. The decision was to have updated breast imaging with a mammogram that showed the breasts were almost entirely fat, and no lesions were seen. We also recommended a breast magnetic resonance imaging (MRI), which was also negative. Due to her family history, she underwent genetic testing, which was negative. Her lifetime risk of developing breast cancer was calculated using the Tyrer-Cuzick model, and it was less than 20%. For this reason, she was not recommended to have additional breast MRIs. Chemoprevention was discussed with the patient and was not recommended.

## Discussion

Ectopic breast tissue found along the embryonic milk lines is rare, occurring in approximately 2%-6% of women and 1%-3% of men, with the axilla being the most frequent location and the vulva being a rarer site [2-4,9]. Variability in presentation depends largely on the degree of breast development along the milk line. Diagnosis requires histological examination, as these lesions may be mistaken for lipomas or malignant lesions. In our case, immunoreactivity for GATA3, estrogen, and progesterone receptors confirmed the breast tissue origin, while negative PAX8 and ERG stains ruled out Müllerian origin and vascular neoplasms, respectively [5,10-12].

While the etiology of ADH within EBT remains unclear, it is more commonly observed in patients who have a strong family history of breast cancer [13,14]. In this case, the patient's positive family history of breast cancer further emphasizes the need for vigilance in patients with similar presentations, including genetic testing. Furthermore, given the morphological similarities between ADH and low-grade DCIS, it is crucial to consider these differentials when working up a vulvar mass [14]. Genetic risk assessment in this case did not reveal any clinically significant variants, suggesting that the ADH was likely sporadic rather than hereditary. There are currently no established guidelines on how to screen, treat, or follow up patients with atypical lesions in EBT. Our treatment approach was to excise the ADH and EBT, which aligns with the standard of care for ADH in the breast [13,15]. The initial workup also included a breast MRI given the identification of a high-risk lesion within the EBT. Although the ADH was located ectopically rather than in the breast, we extrapolated from established breast ADH guidelines and applied the Tyrer-Cuzick model to estimate her lifetime risk. As her calculated risk was <20%, we decided to continue annual mammographic screening without supplemental MRI. Similarly, because the ADH was not located within the breast, and patients with EBT were not considered in the chemoprevention trials, a shared decision was made not to initiate chemoprevention.

This approach underscores both the utility and the limitations of applying conventional breast cancer risk models to ectopic presentations. While such extrapolation provides a pragmatic framework in the absence of evidence-based guidelines, it also highlights the paucity of data on these lesions. Further research in this area is needed and will likely come from case reports and case series, which together can provide the evidence to guide management.

## Conclusions

This case illustrates the diagnostic and management challenges of ADH arising in EBT of the vulva. This case underscores the importance of considering EBT in vulvar masses and highlights the value of histological and clinical correlation in indeterminate tumors that are along the mammary line. Given the rarity of this condition, it is unlikely we will have formal consensus guidelines. Therefore, the management will continue to rely on extrapolation from the management of ADH of the breast and insights from published case reports, with individualized management tailored to each patient.
